# Use of energy dispersive X-ray fluorescence as screening method to detect oregano adulteration

**DOI:** 10.1016/j.crfs.2025.101099

**Published:** 2025-05-29

**Authors:** Sergej Papoci, María Beatriz de la Calle Guntiñas

**Affiliations:** European Commission, Joint Research Centre (JRC), Geel, Belgium

**Keywords:** Oregano, EDXRF, Adulteration, Geographical origin

## Abstract

Oregano is frequently adulterated as demonstrated in a recent control plan organised by the European Commission. In this work, the elemental profiles of 282 oregano samples analysed by energy dispersive X-ray fluorescence were used in combination with multivariate analyses to detect adulteration, in particular with olive leaves. The analyses were carried out in the frame of the coordinated control plan on the authenticity of herbs and spices organised by the European Commission. Partial Least Square Discriminant Analyses, allowed the detection of adulterated samples with a sensitivity of 81 %, and a specificity of 92 %; among the adulterated samples PLS-DA allowed the detection of samples that contained olive leaves with a sensitivity of 94 % and a specificity of 92 %. Copper mass fraction is of relevance because it is significantly higher in samples adulterated with olive leaves. The ratio Cu/Zn allowed the identification of adulterated samples with a sensitivity and specificity of 85 % without the need to use modelling techniques. The elemental profile of oregano obtained by EDXRF was also used to authenticate the geographical origin declared in the labels.

## Highlights

• Substitution of oregano by olive leaves can be detected by EDXRF.
• Oregano adulterated with olive leaves has a higher content of copper.
• The elemental profile of oregano can be used to authenticate its geographical origin.
• SIMCA and PLS-DA are useful modelling tools to detect oregano adulteration.

## Graphical abstract

**Figure:**
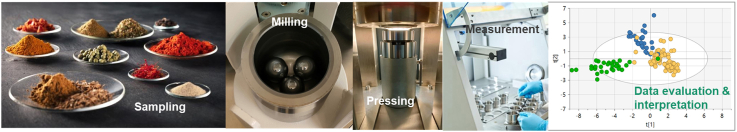

## 1 Introduction

Herbs and spices are not only used for cooking but also as ingredients for pharmaceuticals and food supplements (Maquet et al., 2021). Last years have witnessed an increase in the consumption of herbs and spices in the European Union, triggering an increase in prices that attracts the attention of fraudsters, who profit of the difficulty to control the long and complex, across the world, market of herbs and spices to remain undetected (Van Asselt, Banach, van der Fels-Klerx, 2018). The increase in consumption could be due not only to the use of spices as flavouring healthy ingredients, but also to their health promoting effects. Several articles have been published the last years about oregano phytochemical constituents (Sharifi-Rad et al., 2021; Moghrovyan et al., 2019).

According to the European Parliament resolution of January 14, 2014 on the food crises, fraud in the food chain and the control thereof (European Parliament Resolution, 2014), spices are one of the food commodities most frequently adulterated. For this reason, the European Commission designed a coordinated control plan to detect non-compliances and illegal practices in the herbs and spices sector. Cumin, curcuma, oregano, paprika/chili, pepper and saffron were the herbs and spices included in the control plan. Maybe the most striking outcome of the exercise was that 48 % of the oregano samples analysed were suspicious of being adulterated, mostly with olive leaves.

Different techniques have been used to detect the adulteration of oregano such as, Infra-Red (IR) (Samarco et al., 2023), Fourier Transform-Infra Red (FT-IR) and High Resolution-Liquid Chromatography-Mass Spectrometry (HR-LC-MS) (Black et al., 2016), and NMR (Mandrone et al., 2021).

Commission Regulation (EC) No 149/2008 (Commission Regulation (EC) No 149/2008, 2008) sets maximum residue levels (MRLs) for copper in herbs and spices to be commercialised in the EU. In the particular case of oregano, the MRL is 20 mg kg^−1^. For this reason, in the frame of the analyses carried out by the Joint Research Centre (JRC) for the coordinated control plan on herbs and spices, the copper mass fraction was determined in the included samples to check their compliance with Commission Regulation (EC) No 149/2008. Analyses were carried out by energy dispersive X-ray fluorescence spectroscopy (EDXRF) and the results obtained covered not only the copper mass fraction but also those for other 35 elements. EDXRF was chosen because although characterised by relatively high limits of quantification (LOQs) (at the low mg kg^−1^ for most elements), this technique does not imply the use of hazardous reagents since digestion of the samples is not required, what reduces the price and the environmental impact of the analyses, while increasing the sample throughput.

Elemental profiles obtained by EDXRF have been previously used with success in our laboratory in anti-fraud studies, to authenticate the geographical and botanical origin of honey (Fiamegos, Dumitrascu, Ghidotti, de la Calle Guntiñas, 2020; Ghidotti, Fiamegos, Dumitrascu, de la Calle, 2021), wine (Ghidotti et al., 2024), rice (Dumitrascu, Fiamegos, de la Calle Guntiñas, 2021), coconut sugar (Zdiniakova, de la Calle, 2020), saffron (Ghidotti, Papoci, de la Calle Guntiñas, 2022), and cinnamon, in which also adulteration by substitution with parts of the plant other than the bark was detected (Ghidotti, Papoci, Pietretti, Ždiniaková, de la Calle Guntiñas, 2023).

With the aim to evaluate if the elemental profile of oregano can be used to detect adulteration, especially with olive leaves, multivariate analyses of the data obtained during the control plan were carried out, using Partial Least Square Discriminant Analyses (PLS-DA). A univariate approach is also proposed to detect the adulteration of oregano with olive leaves. The elemental profile of the samples, in combination with two modelling techniques, Soft Independent Modelling of Class Analogy (SIMCA) and PLS-DA was used to authenticate the geographical origin of oregano. This work shows the outcome of those studies, providing sensitivity, and specificity figures for the proposed authentication approaches.

## 2 Materials and methods

### 2.1 Oregano samples

**Fig. 1:**
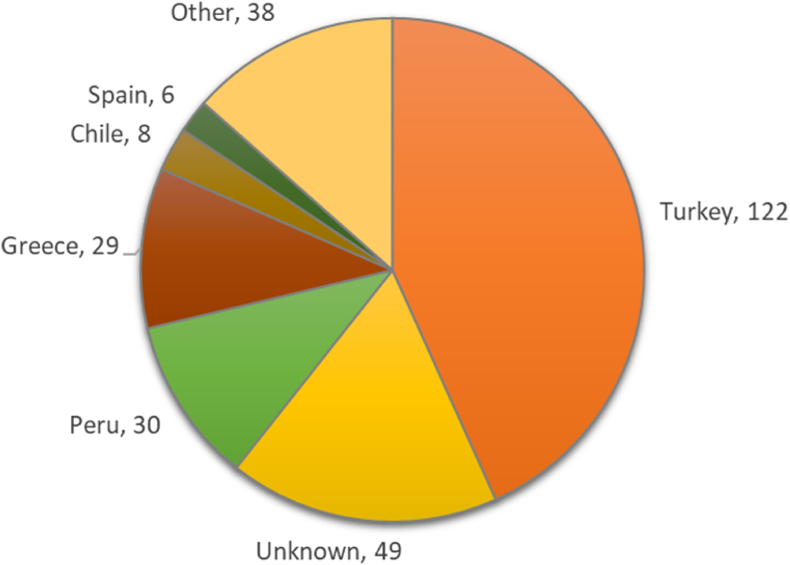
Distribution of analysed samples per country of origin.

Two hundred and eighty-two oregano samples that took part in the EU coordinated control plan to establish the prevalence of fraudulent practices in the marketing of herbs and spices, were analysed by EDXRF. Out of those, 142 were declared not suspicious of adulteration by the different techniques used in the EU coordinated control plan, 79 were found suspicious of containing olive leaves, and 61 were found suspicious of containing other adulterants different from olive leaves. Detailed information about the samples included in the control plan can be found in the report published by the JRC on the outcome of that exercise (Maquet et al., 2023). Summarising, around 80 % of the samples were sampled by competent Member States (MSs) authorities, at producers, control posts, importers, wholesalers, and storage/processing/packaging establishments, and around 20 % at distribution and retail level. Twenty-three MSs of the European Union plus Norway and Switzerland submitted samples for the study. The distribution per country of origin is shown in Fig. 1; twenty-two countries sent five or less samples and are included in Fig. 1 as “Other”.

### 2.2 Reagents and standards

Deionised water from a Milli-Q Plus system (>18.3 MΩ) (Millipore, Billerica, MA, USA) was used to clean the material used in sample processing, with the aim of avoiding cross-contamination.

The reference sample FLX-S13 (Fluxana, Bedburg-Hau, Germany) was used to check the performance of the EDXRF spectrometer.

The calibration curves used to quantify major- and trace elements by EDXRF, were constructed with 45 certified reference materials (CRMs) and reference materials (RMs), 23 of them had an organic matrix. Another 25 CRMs and RMs, 13 out of them with an organic matrix, were used to evaluate the accuracy of the method. The list of organic and inorganic CRMs used for calibration purposes and to test the method accuracy are given in Table 1.

**Table 1:** LOQ of the EDXRF method, expanded uncertainty (U)(k = 2) associated to the results, and information about the results obtained in the oregano samples for: **a)** Mg, Al, Si, P, Cl, S, K, Ca, Ti, Cr, Mn, Fe, Ni, Cu, Zn, As, Br, Rb, and **b)** Sr, Zr, Nb, Cd, Ba, Pb, Hg, V, Co, Se, Mo, Sn, Sb, Cs, La, Sm, Ce, Nd.

a)																		
ID	Mg	Al	Si	P	Cl	S	K	Ca	Ti	Cr	Mn	Fe	Ni	Cu	Zn	As	Br	Rb
**LOQ** (mg kg^−1^)	*1450*	*860*	*2348*	*171*	*78*	*700*	*566*	*118.4*	*302*	*1.89*	*2.55*	*4.6*	*0.16*	*1.2*	*5.8*	*1.01*	*1.7*	*4.2*
**U (%)(k = 2)∗**	*13*	*17*	*12*	*6*	*2*	*10*	*3*	*3.5*	*10*	*20*	*11*	*6.5*	*25*	*10.5*	*6.5*	*15*	*22*	*5*
***Oregano samples without olive leaves***																		
**Minimum** (mg kg^−1^)	< LOQ	< LOQ	< LOQ	967.2	260.6	1511	9673	11213	< LOQ	< LOQ	11.96	108.1	0.26	5.24	17.45	< LOQ	< LOQ	< LOQ
**Maximum** (mg kg^−1^)	4925	2766	3427	3872	9675	5774	36119	21351	< LOQ	19.03	278.8	1887	16.11	101.6	69.35	4.22	39.10	41.33
**Median** (mg kg^−1^)	2820	< LOQ	< LOQ	1695	1027	2562	16577	16886	< LOQ	3.26	57.41	509.8	5.23	8.90	26.73	< LOQ	4.79	15.68
**Mean** (mg kg^−1^)	2966	< LOQ	< LOQ	1814	1724	2612	17849	16434	< LOQ	3.93	66.18	561.1	4.87	9.83	28.33	< LOQ	7.94	15.78
**STD** (mg kg^−1^)	704.1			402.0	1631	554.4	3904	2076		3.24	33.20	271.4	3.10	6.99	6.83		8.24	5.51
**STD** (%)	23.7			22.2	94.6	21.2	21.9	12.6		82.4	50.2	48.4	63.7	71.2	24.1		103.7	34.9
***Oregano samples with olive leaves***																		
**Minimum** (mg kg^−1^)	1725	< LOQ	< LOQ	579.4	282.9	1111	7545	12530	< LOQ	< LOQ	31.31	150.8	1.18	5.22	13.59	< LOQ	< LOQ	< LOQ
**Maximum** (mg kg^−1^)	4711	4191	5513	1929	3588	3803	18222	21766	< LOQ	17.50	187.7	2177	12	56.86	52.58	1.07	14.33	25.91
**Median** (mg kg^−1^)	2693	< LOQ	< LOQ	921.3	568.1	1545	9620	16623	< LOQ	2.45	48.07	311.3	2.61	23.22	18.7	< LOQ	3.92	7.52
**Mean** (mg kg^−1^)	2761	< LOQ	< LOQ	986.8	799.6	1680	10440	16647	< LOQ	3.36	50.14	416.8	3.31	23.74	20.42	< LOQ	4.21	8.21
**STD** (mg kg^−1^)	516.9			278.4	662.8	446.7	2425	2025		2.74	19.72	330.4	2.02	12.27	5.84		2.58	3.73
**STD** (%)	18.7			28.2	82.9	26.6	23.2	12.2		81.5	39.3	79.3	61.1	51.7	28.6		61.3	45.5

b)																		
ID	Sr	Zr	Nb	Cd	Ba	Pb	Hg	V	Co	Se	Mo	Sn	Sb	Cs	La	Sm	Ce	Nd
**LOQ** (mg kg^−1^)	*1.19*	*10.09*	a	*0.7*	*2.4*	*1*	*1.7*	*4.12*	*0.7*	*0.365*	*0.41*	*2.5*	*0.87*	*3.2*	*2.3*	*3.3*	*4.18*	*18.4*
**U (%)(k = 2)∗**	*8*	*5*	a	*25*	*18*	*20*	*20.5*	*15*	*10*	*9*	*15*	*6*	*17.5*	*5*	*3*	*17.5*	*4*	*10*
***Oregano samples without olive leaves***																		
**Minimum** (mg kg^−1^)	3.26	< LOQ	0.17^a^	< LOQ	7.37	< LOQ	< LOQ	< LOQ	< LOQ	< LOQ	< LOQ	< LOQ	< LOQ	< LOQ	< LOQ	< LOQ	< LOQ	< LOQ
**Maximum**(mg kg^−1^)	126.4	11.21	1.02^a^	< LOQ	47.46	5.25	3.61	4.92	1.51	0.95	1.92	< LOQ	1.17	3.17	< LOQ	< LOQ	< LOQ	< LOQ
**Median** (mg kg^−1^)	27.09	< LOQ	0.50^a^	< LOQ	18.42	< LOQ	< LOQ	< LOQ	< LOQ	0.43	< LOQ	< LOQ	< LOQ	< LOQ	< LOQ	< LOQ	< LOQ	< LOQ
**Mean** (mg kg^−1^)	40.06	< LOQ	0.52^a^	< LOQ	19.54	< LOQ	< LOQ	< LOQ	< LOQ	0.44	0.46	< LOQ	< LOQ	< LOQ	< LOQ	< LOQ	< LOQ	< LOQ
**STD** (mg kg^−1^)	27.40		0.16		6.07					0.14	0.33							
**STD (%)**	68.4		30.4		31.1					33.0	70.8							
***Oregano samples with olive leaves***																		
**Minimum** (mg kg^−1^)	12.83	< LOQ	a	< LOQ	7.41	< LOQ	< LOQ	< LOQ	< LOQ	< LOQ	< LOQ	< LOQ	< LOQ	< LOQ	< LOQ	< LOQ	< LOQ	< LOQ
**Maximum** (mg kg^−1^)	162.1	16.56	a	< LOQ	35.1	1.67	2.04	5.53	1.54	0.72	0.44	< LOQ	1.18	< LOQ	2.87	< LOQ	< LOQ	< LOQ
**Median** (mg kg^−1^)	42.55	< LOQ	a	< LOQ	16.15	< LOQ	< LOQ	< LOQ	< LOQ	0.38	< LOQ	< LOQ	< LOQ	< LOQ	< LOQ	< LOQ	< LOQ	< LOQ
**Mean** (mg kg^−1^)	51.41	< LOQ	a	< LOQ	16.73	< LOQ	< LOQ	< LOQ	< LOQ	0.38	< LOQ	< LOQ	< LOQ	< LOQ	< LOQ	< LOQ	< LOQ	< LOQ
**STD** (mg kg^−1^)	27.69				4.81					0.13								
**STD (%)**	53.9				28.7					32.6								

a Semi-quantitative data provided by the EDXRF software ε5.

### 2.3 Sample preparation

To carry out the analyses, 6 g of oregano were milled using a Planetary MonoMill-Minimill II of Fritsch-PANalytical (Almero, The Netehrlands), using a 250 mL tungsten carbide grinding bowl with 5 tungsten carbide grinding balls (30 mm diameter). The milling time was adapted till no coarse particles were observed, in all cases it was more than 6 min. Forty millimetres diameter pellets were made pressing the 6 g of milled oregano at 250 kN for 3.5 min, with a semi-automatic press from Hertzog Machinenfabrik Gmbh (Osnabrück, Germany). Once obtained, the pellets were directly analysed by EDXRF without any further treatment.

### 2.4 Instrumentation

The elemental profile of the oregano samples was determined by EDXRF with an Epsilon 5 spectrometer (PANalytical, Almerlo, The Netherlands), with a method previously validated using the CRMs and RMs listed in Supplementary 1. Detailed information about measurement parameters, and the validation approach has been already published elsewhere (Fiamegos, de la Calle Guntiñas, 2018). The LOQ and expanded Uncertainty (k = 2) of the method are provided in Table 1. No validation of the method could be carried for the analyses of Nb due to the lack of CRMs and RMs with certified values for that element. The semi-quantitative results provided for that element by the ε5 software were used for modelling purposes.

The performance of the EDXRF was checked every week making use of the reference sample FLX-S13, following the instrument manufacturer's instructions, to correct the normal drift of the spectrometer. No systematic bias was detected during the period in which the measurements were carried out.

### 2.5 Uni- and multivariate analyses

The following elements were analysed in all the oregano samples: Mg, Al, Si, P, Cl, S, K, Ca, Ti, Cr, Mn, Fe, Ni, Cu, Zn, As, Br, Rb, Sr, Zr, Nb, Cd, Ba, Pb, Hg, V,Co, Se, Mo, Sn, Sb, Cs, La, Sm, Ce and Nd.

Student's t-tests, used to determine which elements were present at significantly different mass fractions in adulterated and non-adulterated oregano, and in oregano with and without olive leaves, were run using the software Statistica version 13.0.5.17 (TIBCO, Software Inc.).

Multivariate analyses of the elemental mass fractions were carried out with the software SIMCA® version 17 (Umetrics, Malmö, Sweden) (Eriksson et al., 2013).

The mass fractions of all the elements analysed in the oregano samples were used for modelling purposes, including those below the LOQ of the method, because although the mass fractions of some elements were too low to be quantified in some or all of the samples, they were high enough to be detected, contributing to an improvement in the performance of the models. The raw data obtained from the EDXRF instrument were used for modelling purposes.

Principal component analysis (PCA), a non-supervised multivariate analysis tool, and PLS-DA, a supervised multivariate analysis tool, were used to visualise the projection of the samples in function of their elemental profiles. The amount of principal components was always kept to three to void overfitting. Mass fractions were normalised by uni-variance scaling, and no further pre-processing was applied.

PLS-DA, a supervised multivariate analysis tool that maximises the differences between categories, was used to classify the oregano samples as suspicious (S) or non-suspicious (NS) of adulteration with straneous species, and as S or NS of containing olive leaves. The two categories were defined making use of the results obtained during the control plan exercise using DNA, IR and HR-LC-MS based methods.

SIMCA, a supervised multivariate analysis tool that maximises the similarities among observations within a category, followed by PLS-DA were used to authenticate the geographical origin of the oregano samples as declared on the labels.

All analysed elements and all samples in a certain category (NS (n = 142), S with olive leaves (n = 79), and S without olive leaves (n = 61)), were used for modelling purposes; the amount used in the models for geographical origin authentication can be found in Table 2. The validation of the different models was done with the leave-one-out cross validation method, because splitting the datasets in two, one for training and another one for validation, would have resulted in small sample sets in the geographical origin authentication studies; preference was given to the use of one single validation approach throughout the study. The sensitivity, specificity, and accuracy of the classification studies were calculated using the approach proposed by Barbosa et al. (Barbosa et al., 2016).

**Table 2:** Sensitivity, specificity and accuracy of different modelling approaches to authenticate the geographical origin of oregano.

Country	N	TP	FN	Sensitivity	N	TN	FP	Specificity	Accuracy
SIMCA									
**Turkey**	122	106	16	**87**	85	77	8	**91**	**88**
**Greece**	29	26	3	**90**	177	166	11	**94**	**93**
**Perú**	30	27	3	**90**	176	169	7	**96**	**95**
**SIMCA followed by PLS-DA**									
**Turkey**	122	110	12	**90**	85	77	8	**91**	**90**
**Greece**	29	27	2	**93**	177	169	8	**95**	**95**
**Perú**	30	29	1	**97**	176	171	5	**97**	**97**

## 3 Results and discussion

### 3.1 Results of the elemental analyses by EDXRF

The results of the elemental determination by EDXRF are shown in Supplementary 1. With some exceptions, probably due to some extreme outliers, there is a good agreement between the median and the mean, demonstrating that the results are normally distributed. It was observed that for most elements, the median and mean of the samples without olive leaves is higher than those in the samples with olive leaves. There are only three exceptions to that rule, copper, strontium and lead, although in the case of lead most mass fractions are below the LOQ of the method for that element and results can only be taken as indicative. In the case of copper, the median and the mean in the samples containing olive leaves are around three times higher than in the samples without olive leaves.

The *t*-test's showed that among the elements with concentrations in most or in all samples higher than their respective LOQ, Mg, P, Cl, S, K, Mn, Fe, Ni, Cu, Zn, As, Br, Rb, Sr and Ba had mass fractions significantly different in the oregano samples with and without olive leaves, respectively.

Commission Regulation (EC) No 149/2008 sets an MRL for copper in oregano of 20 mg kg^−1^. Forty-eight of the analysed samples had copper mass fractions higher than the allowed MRL, 46 out of them (96 %) were samples that contained olive leaves. This is probably due to the use of copper containing pesticides on olive trees.

### 3.2 Detection of adulteration with extraneous species. The special case of olive leaves

#### 3.2.1 Detection of adulteration using multivariate analyses and modelling

Among the different modelling tools that can be applied in authentication studies, maybe two of the more widely used are SIMCA and PLS-DA, both supervised techniques. SIMCA, that maximises the similarities among observations within a category, is not a discriminant tool and was developed for the purpose of characterising a certain category (Rácz, Gere, Bajusz, H'eberger, 2018), and for this reason its performance for classification purposes is not as good as that obtained with other classification tools such as PLS-DA. PLS-DA, is a supervised technique that maximises the differences among categories. A drawback of PLS-DA is that it forces the classification of an observation in one of the categories used to build-up the model. Nevertheless, this is not a problem in this study because all oregano samples are either adulterated or non-adulterated. For this reason, PLS-DA was used to discriminate non-adulterated oregano samples from those that are adulterated. The validation of the PLS-DA model was carried out leaving each time one sample out and classifying it with the model constructed with the remaining samples. The sensitivity of the model was 81 %, with 112 true positives (TP), samples correctly classified as being adulterated, and 26 false negatives (FN), adulterated samples wrongly classified as non-adulterated. The method had a specificity of 92 %, with 132 true negatives (TN), samples correctly classified as non-adulterated, and 12 false positives (FP), non-adulterated samples wrongly classified as adulterated. Two out of the 12 FP were extreme outliers for the Cu mass fractions, with Cu contents close to 40 and 100 mg kg^−1^, respectively. Those two samples are legally non-compliant with Commission Regulation (EC) No 149/2008 on maximum residue levels, and only for that reason they should not enter the EU market.

An attempt was made to improve the sensitivity of the PLS-DA model using exclusively as variables the elements with significantly different mass fractions in adulterated and non-adulterated samples, respectively, but no significant improvement was observed.

**Fig. 2:**
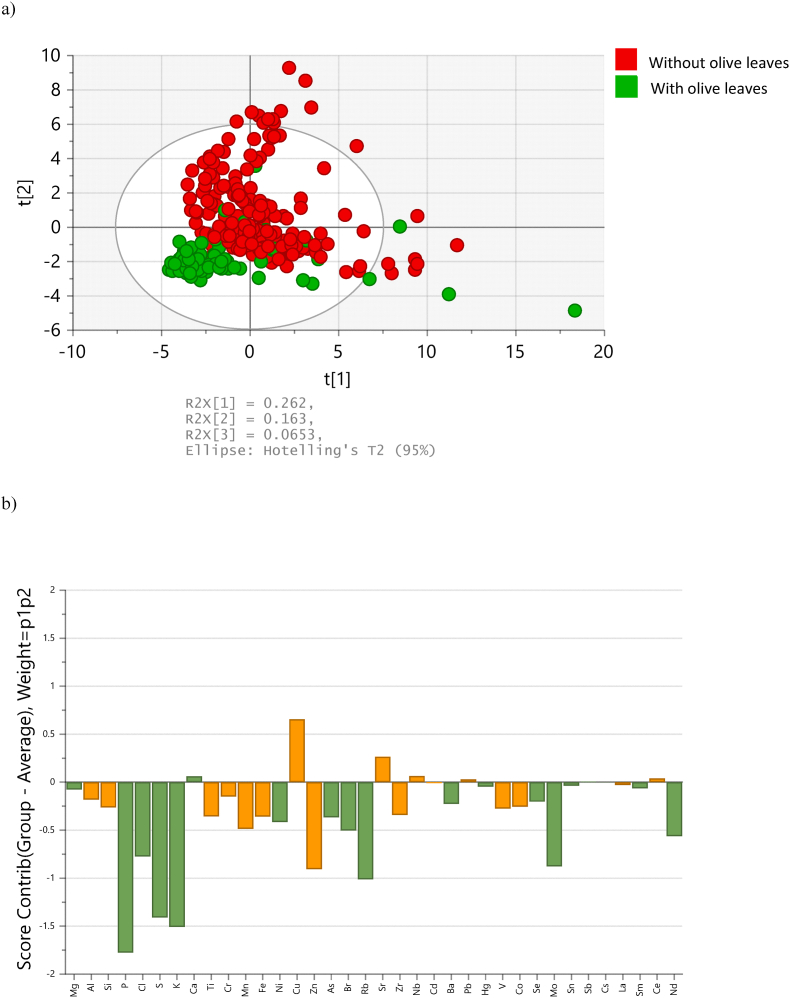
**a)** PCA score plot of all the oregano samples analysed, and **b)** score contribution to the PCA score plot of all the oregano samples analysed, of the different elements included in the study.

The PCA score plot of all the oregano samples analysed, Fig. 2a, showed that most of the samples adulterated with olive leaves form a cluster separated from those that do not contain olive leaves. Since adulteration of oregano with olive leaves is probably the most frequent type of adulteration of oregano, a second PLS-DA model was constructed to differentiate the samples adulterated with olive leaves from samples containing other type of adulterant. This second PLS-DA model was characterised by a sensitivity of 94 % (5 FN) and a specificity of 92 % (9 FP). The samples adulterated with olive leaves and that were not detected as suspicious of adulteration by the first PLS-DA model, and as suspicious of adulteration with olive leaves by the second model, were those with a very low content of olive leaves, according to the HPLC-MS analyses (Maquet et al., 2023).

#### 3.2.2 Detection of the presence of olive leaves in oregano through the copper mass fraction. Univariate approach

Since the group of oregano samples containing olive leaves has on average three times more Cu than the samples without olive leaves, an attempt was made to detect the adulterated samples through the Cu mass fraction using a univariate approach.

**Fig. 3:**
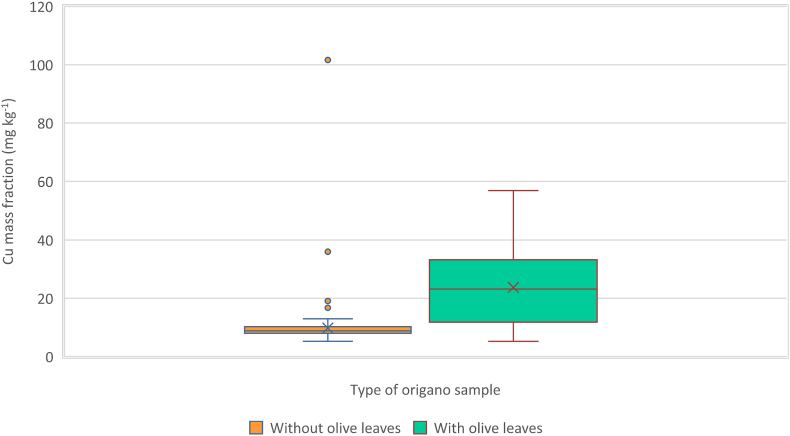
Cu mass fractions Box and whisker plot of the oregano samples with and without olive leaves, respectively.

**Fig. 4:**
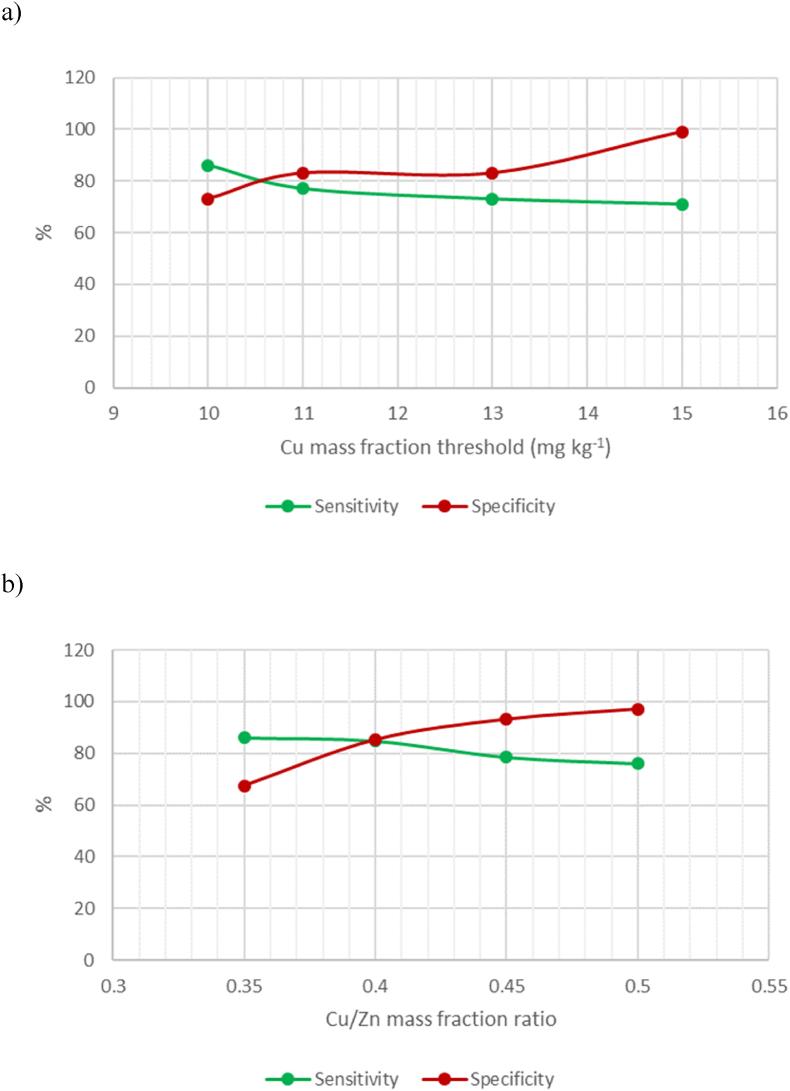
Optimisation of the classification threshold using: **a)** Cu mass fraction, and **b)** Cu/Zn ratio, as univariate approach to detect the presence of olive leaves in oregano.

As shown by the box and whisker plot, Fig. 3, the Cu mass fraction ranges of the two groups of samples, with and without olive leaves, overlap. For this reason, a Cu content classification threshold has to be found that provides optimum sensitivity and specificity. As shown in Fig. 4a, such a threshold was 10.5 mg kg^−1^, which resulted in a sensitivity of 81 %, and a specificity of 78 %.

Fig. 2b shows that the oregano samples that contain olive leaves, are not only particularly rich in Cu, but are also particularly poor in Zn when compared to the samples without olive leaves. For this reason, a study was run to try to improve the accuracy of the classification making use of the ratio Cu/Zn. As expected, both sensitivity and specificity improved, reaching a value of 85 % for both parameters with a threshold for the Cu/Zn ratio of 0.4, Fig. 4b.

The described univariate approach could be used by laboratories that are not familiar with multivariate analyses and modelling.

### 3.3 Authentication of oregano geographical origin

**Fig. 5:**
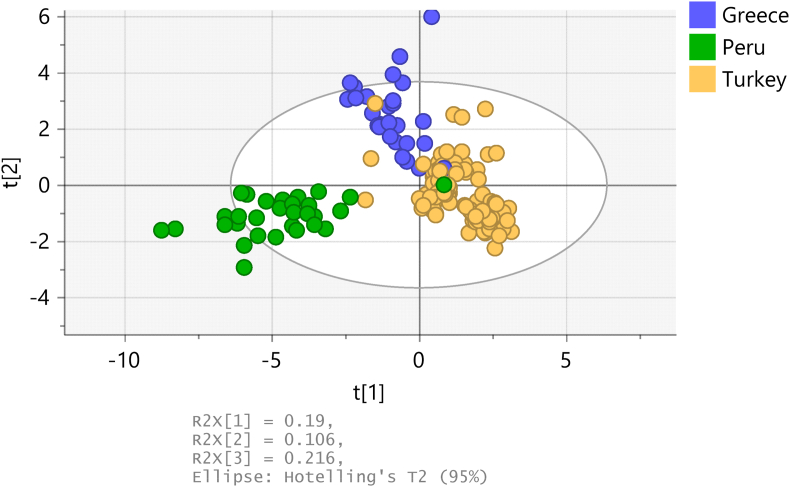
PLS-DA score plot of the Turkish, Greek and Peruvian oregano samples, according to country of origin.

Plants take up elements from the ground in which they grow, and for that reason their elemental profiles have been used (alone or in combination with other compounds) followed by multivariate analyses to authenticate their geographical origin (Kelly et al. (2002); Dumitrascu et al. (2021); Ghidotti et al. (2024); Wakefield et al. (2019)). Fig. 5 shows that the oregano samples from Turkey, Greece and Peru form three distinct clusters in a PLS-DA score plot.

The modelling approach used to authenticate the geographical origin of oregano was different than the one chosen to classify oregano as pure or adulterated, and the adulterated oregano as containing olive leaves or not. Those models could be constructed with two categories, adulterated/non-adulterated, with olive leaves/without olive leaves, which is the ideal situation for PLS-DA. The authentication of the geographical origin is more complex because the amount of categories should be as large as countries in which oregano grows. An initial attempt was made to use PLS-DA models constructed with two categories, for instance Turkish/non-Turkish, Greek/non-Greek, Peruvian/non-Peruvian, etc; however, the standard deviation of the composition of the non-xxx category was very large in comparison with that of the Turkish, Greek, Peruvian, categories, resulting in a poor performance of the models. Therefore, SIMCA was considered a better option because it does not force the classification of a sample in one of the categories included in the model, evaluating only if a sample is similar or not to the samples used to train the model for a certain category.

In this study, 64 % of the samples came from Turkey, Greece and Peru, and in order to have a relatively high amount of samples per category, the study to test if the elemental composition of oregano obtained by EDXRF can be used to authenticate the geographical origin of a sample, was limited to those three categories. The sensitivities of the SIMCA model, evaluated leaving one sample out, are given in Table 2, and varied from 87 % for the Turkish oregano, to 90 % for the Greek and Peruvian.

Oregano is native to Mediterranean and Western Asian countries, growing also in some areas in America. Only samples coming from those regions according to the label declarations, were included in the specificity study. The specificity of the SIMCA model varied in the range 91–96 %, Table 2.

In case a sample labelled as Turkish, Greek or Peruvian, was classified by SIMCA as belonging to one of the other two categories, the sample was classified in a second step using a PLS-DA model constructed for the same three categories. By doing so, the sensitivities improved and varied from 90 to 97 %, and specificity from 91 to 97 %. The approach of using a two-step classification, first with SIMCA and then with a PLS-DA model, has been successfully used to classify honey samples according to botanical origin (Fiamegos et al., 2020). Unfortunately, the second classification by PLS-DA could not be used in the specificity study to classify samples that according to their labels do not come from Turkey, Greece, or Peru, because PLS-DA would force their classification as belonging to the category with elemental profiles more similar to the one of the sample in question. For instance, Chilean or Bolivian oregano would be very likely classified as Peruvian, and Italian and Spanish oregano as Greek or Turkish.

## 4 Conclusions

This work demonstrates that EDXRF is a suitable screening method to detect oregano adulteration with other species, and to authenticate the geographical origin of the product. The method is clean and characterised by a high sample throughput because it does not require sample digestion, reducing in this way the cost of the analyses. For those reasons, the approach is ideal to be used by control laboratories.

SIMCA allowed the authentication of the geographical origin of oregano. The performance of the authentication could be improved with a combination of SIMCA with PLS-DA that provides sensitivities and specificities higher than 90 %. However, a database well populated with results obtained with samples coming from all the main producing countries, would be needed.

## CRediT authorship contribution statement

**Sergej Papoci:** Investigation. **María Beatriz de la Calle Guntiñas:** Conceptualization, Methodology, Formal analysis, Writing – original draft, Visualization, Supervision, Project administration.

## Declaration of competing interest

The authors declare that they have no known competing financial interests or personal relationships that could have appeared to influence the work reported in this paper.

## Data Availability

Data will be made available on request.
