# Supplementary material for: Use of energy dispersive X-ray fluorescence as screening method to detect oregano adulteration
Source: Curr Res Food Sci. 2025 May 29;10:101099. doi: 10.1016/j.crfs.2025.101099 (PMC12169713; doi:10.1016/j.crfs.2025.101099)
Supplement: Multimedia component 1 [file mmc1.docx]

**Supplementary 1:** List of CRMs and RMs used in the validation of the EDXRF method for calibration purposes, and in accuracy studies.

| ***Organic CRMs and RMs used for calibration purposes*** | | | | | |
| --- | --- | --- | --- | --- | --- |
| **CRM/RM** | **Matrix** | **CRM/RM** | **Matrix** | **CRM/RM** | **Matrix** |
| OBTL-5 | Tobacco | ERM-CD-281 | Rye grass | BCR-129 | Hay powder |
| NIST 1567b | Wheat flour | IAEA-359 | Cabbage | BCR-679 | White cabbage |
| NIST 1570a | Spinach leaves | NIST 1573a | Tomato leaves | NIST 1575a | Pine needles |
| GSV1 | Bush leaves | GSV2 | Bush leaves | GSV3 | Poplar leaves |
| GSV4 | Tea leaves | NBS 1571 | Orchard leaves | NBS 1572 | Citrus leaves |
| ERM-CD-200 | Algae | IAEA-392 | Algae | IAEA-413 | Algae |
| BCR-482 | Lichen | NMIJ CRM7405-a | Algae | IAEA-336 | Lichen |
| EURL-HM-22 | Fish | GSH 1 | Human hair |  |  |
| ***Organic CRMs and RMs used for accuracy studies*** | | | | | |
| **CRM/RM** | **Matrix** | **CRM/RM** | **Matrix** | **CRM/RM** | **Matrix** |
| BRAN-1 | Corn bran | DUWF-1 | Durum wheat flour | SOWW-1 | Soft winter wheat flour |
| NIST 1568b | Rice flour | BCR-191 | Brown bread | PVTL-6 | Tobacco |
| RT3* | Tobacco | RT5* | Tobacco | AJJA 17* | Tobacco |
| ERM-BD150 | Skimmed milk | ERM-CE278IK | Mussel tissue | ERM-CE464 | Tuna fish |
| IMEP-119 | Vegetable feed |  |  |  |  |

* Reference materials characterised by several laboratories using ED-XRF, ICP-MS, and k0-NAA.
